# Communication about vaccine efficacy and COVID-19 vaccine choice: Evidence from a survey experiment in the United States

**DOI:** 10.1371/journal.pone.0265011

**Published:** 2022-03-30

**Authors:** Sarah Kreps, Douglas L. Kriner

**Affiliations:** Department of Government, Cornell University, Ithaca, NY, United States of America; The University of Jordan, JORDAN

## Abstract

While mass vaccination campaigns against COVID-19 have inoculated almost 200 million Americans and billions more worldwide, significant pockets of vaccine hesitancy remain. Research has firmly established that vaccine efficacy is an important driver of public vaccine acceptance and choice. However, current vaccines offer widely varying levels of protection against different adverse health outcomes of COVID-19. This study employs an experiment embedded on a survey of 1,194 US adults in June 2021 to examine how communications about vaccine efficacy affect vaccine choice. The experiment manipulated how vaccine efficacy was defined across four treatments: (1) protection against symptomatic infection; (2) protection against severe illness; (3) protection against hospitalization/death; (4) efficacy data on all three metrics. The control group received no efficacy information. Subjects were asked to choose between a pair of vaccines—a one-dose viral vector vaccine or two-dose mRNA vaccine—whose efficacy data varied across the four experimental treatment groups. Efficacy data for each vaccine on each dimension were adapted from clinical trial data on the Johnson & Johnson/Janssen and Pfizer/BioNTech vaccines. Among all respondents, only modest preference gaps between the two vaccines emerged in the control group and when the two vaccines’ roughly equivalent efficacy data against hospitalization and death were reported. Strong preferences for a two-dose mRNA vaccine emerged in treatments where its higher efficacy against symptomatic or severe illness was reported, as well as in the treatment where data on all three efficacy criteria were reported. Unvaccinated respondents preferred a one-dose viral vector vaccine when only efficacy data against hospitalization or death was presented. Black and Latino respondents were significantly more likely to choose the one-shot viral vector vaccine in the combined efficacy treatment than were whites. Results speak to the importance of understanding how communications about vaccine efficacy affect public preferences in an era of increasing uncertainty about efficacy against variants.

## 1. Introduction

Severe acute respiratory syndrome coronavirus 2 (SARS-CoV-2, otherwise known as COVID-19) has been linked to close to 200 million cases and 4 million deaths as of July 2021 [1]. For most of the first year of the pandemic, cases, hospitalizations, and deaths tracked closely, with cases acting as a leading indicator of both hospitalizations and fatalities. With the introduction of COVID-19 vaccines, the relationship between cases and both hospitalizations and fatalities has begun to diverge in some contexts. In the UK, for example, by July 2021 about 70% of the population had received at least one dose whereas 50% had received two doses of a vaccine. However, COVID-19 cases continued to climb to levels last seen in late January 2021 when the country was still experiencing its post-holiday wave. Deaths, by contrast, remained low, down almost 99% from their early 2021 peak [2]. This reflects the sometimes significant variation in vaccine effectiveness at preventing symptomatic illness, versus severe disease, versus hospitalization and death. This real-world data mirrors evidence from clinical trials showing that the Oxford AstraZeneca vaccine, which has been used extensively in the UK, is significantly more effective at protecting against hospitalization and death than against symptomatic infection [3].

Efficacy is often cited as the most relevant determinant of individual inclination to vaccinate both in general [4–6] and for the COVID-19 vaccine in particular [7–10]. However, vaccine efficacy is measured with respect to different outcomes, and different media outlets often emphasize different efficacy figures, potentially confusing the public. For example, when Johnson & Johnson released efficacy data for the Janssen COVID-19 clinical trials in February 2021, some media outlets emphasized its 66% efficacy against symptomatic infection–a figure significantly lower than those reported for vaccines manufactured by Pfizer and Moderna. Other outlets focused on its 85% efficacy against severe forms of COVID-19, while others emphasized its near 100% efficacy against hospitalization and death. Recognizing the importance of how the media frames vaccine efficacy, an article in *Forbes* led with the title “Johnson & Johnson Vaccine’s 66% Efficacy Figure is Better than it Looks” [11].

The near-ubiquity of efficacy as a key factor in studies of vaccine preferences suggests that communication about efficacy—protection against illness versus hospitalization versus death—may affect attitudes about vaccines and in turn vaccine choice. For example, in the United States the one-dose Janssen COVID-19 vaccine has been cited as appealing in principle especially for homeless populations that are transient and harder to track for a second shot [12] and Hispanics who express concerns about taking time off for vaccination or side effects [13]. Yet many media accounts that reported vaccine efficacy levels as protection against symptomatic illness rather than hospitalization and death—sometimes without specifying which of these two outcomes—implied that the one-dose viral vector vaccine was less desirable than the two-dose mRNA alternative [14]. As new vaccines enter the market, efficacy communications could similarly affect public acceptance depending on how the reported efficacy figures of those vaccines compare to those of the first mRNA vaccines.

This study employs an original survey experiment to examine how communications surrounding COVID-19 vaccine efficacy levels influence public vaccine preferences. The experiment examines how public attitudes toward two hypothetical vaccines—with characteristics modeled on two currently available vaccines—change depending on whether information provided about efficacy is defined as protection against symptomatic infection, severe illness, or hospitalization and death. Because efficacy communications may be critical to combatting continued vaccine hesitancy, in addition to assessing aggregate effects among all subjects, the analysis also examines the effects of different efficacy communications on the preferences of unvaccinated subjects and across racial/ethnic subgroups.

### 1.1 Vaccine characteristics and public willingness to vaccinate

A growing literature has examined how different vaccine attributes influence public acceptance in the United States and around the world. While some of the findings conflict across countries and even within countries from study to study, one near universal finding is that efficacy is one of the most important predictors of public vaccine acceptance and choice [7,9,10,15–17]. All else equal, when offered a choice between two vaccines, people are more likely to choose the vaccine with higher reported efficacy. However, existing research suggests that the size of this preference gap may vary with the size of the efficacy gap between the vaccines. For example, a series of studies employing conjoint experiments in the United States found that a large preference gap for a vaccine that was 90% effective versus one that was 50% effective, but the preference gap between the former and a vaccine that was 70% effective was considerably smaller [7,9], and in one case the difference was not statistically significant [10]. In nearly all of these studies, efficacy is underspecified. In the context of COVID-19 vaccines, the type of efficacy—against symptomatic illness versus severe disease versus hospitalization and death—has varied widely and we know little about how different operationalizations of efficacy affect preferences toward individual vaccines.

Moreover, past research has identified a range of factors that also influence vaccine preferences and choice, including the prevalence of side effects [7,9,10,15–17], the national origin of the vaccine [7–9,16,18], the nature of the governmental approval process [7,18,19], and endorsements [7,20–22], among others. Two characteristics of the vaccine itself that may also influence public acceptance and choice are the technology underlying the vaccine and the number of doses required.

Vaccines developed by Pfizer/BioNTech and Moderna both employed novel mRNA technology; the two vaccines were the first using mRNA to receive approval or authorization from federal regulators. In part because of the pioneering nature of the technology, online misinformation about mRNA vaccines abounds, including the false claim that these vaccines can alter recipients’ DNA [23]. Misinformation has stoked vaccine hesitancy [23], though some interventions may be able to counteract or at least blunt the adverse effects [24,25]. Research has shown that exposure to this specific false claim can significantly reduce public willingness to vaccinate against COVID-19 [26]. Other COVID-19 vaccines including those developed by AstraZeneca and Johnson & Johnson/Janssen use more established viral vector technologies.

Several studies have examined the influence of vaccine type on public preferences. An analysis of public opinion in Israel showed greater hesitancy to an mRNA vaccine [27], though an analysis of students in Italy suggested greater hesitancy toward a viral vector reverse [28]. On the other hand, a study in the United States showed little evidence that vaccine type significantly influenced vaccine choice [9].

Another feature of COVID-19 vaccines with immediate practical and logistical implications is the number of doses required. Most currently available vaccines including those developed by Pfizer/BioNTech, Moderna, and AstraZeneca require two doses administered at an interval of at least three weeks. The vaccine developed by Johnson & Johnson/Janssen received FDA Emergency Use Authorization in the United States as a single-shot regimen. Several studies suggest that a two-dose regimen decreases vaccination intention, though the size of the effect varied across studies and contexts [9,21,27].

In the experiment that follows, we ask subjects to choose between a pair of hypothetical vaccines. The first is a one-dose, viral vector vaccine; the second is a two-dose, mRNA vaccine. These basic parameters reflect the characteristics of the three vaccines currently authorized for use in the United States. An experimental control group allows us to establish a baseline of public preferences between these two types of vaccines absent information about their relative efficacy. A series of treatment groups presenting subjects with different communications about relative vaccine efficacy examines how vaccine choices change depending on how efficacy is defined and the size of the efficacy gap between the alternatives.

## 2. Materials and methods

### 2.1. Data

Our survey protocol was approved by Cornell University’s Institutional Review Board (IRB #2004009569). All survey respondents provided informed written consent before beginning the survey. Our survey sample was indirectly recruited via the Lucid online marketplace. On June 28, 2021, Lucid contacted 2,422 US adults to recruit a convenience sample of 1,200 participants who consented to begin our survey containing an embedded survey experiment (1,194 completed the survey). This sample size per experimental treatment group allows the detection of differences in means of 12% with a type I error rate of α = .05 and a power of .80. Lucid uses quota sampling to produce samples matched to the demographics of the U.S. population on age, gender, ethnicity, and geographic region. Research has shown that experimental effects observed in Lucid samples largely mirror those found using probability-based samples [29]. Across our sample, 801 subjects reported being vaccinated, and 393 were unvaccinated. Full sample demographics are reported in Table 1. Comparisons of the sample demographics to those of other prominent social science surveys and U.S. Census figures are shown in S1 Table.

**Table 1:** Survey sample demographics.

	N	Percentage
**Age**		
18–29	258	(21%)
30–44	357	(30%)
45–59	284	(24%)
> = 60	295	(25%)
**Gender**		
Male	629	(53%)
Female	565	(47%)
**Race/Ethnicity**		
White	881	(74%)
Black	153	(13%)
Latino	113	(9%)
Asian	70	(6%)
**Education**		
Less than High School	26	(2%)
High School / GED	305	(26%)
Some College	405	(34%)
4-Year College Degree	265	(22%)
Graduate School	193	(16%)
**Income**		
< $20,000	267	(22%)
$20,000 to $39,999	271	(23%)
$40,000 to $59,999	222	(19%)
$60,000 to $79,999	153	(13%)
$80,000 to $99,999	97	(8%)
> = $100,000	184	(15%)
**Political Partisanship**		
Democrat (includes leaners)	605	(51%)
Republican (includes leaners)	405	(34%)
Independent	184	(15%)
**Vaccination Status**		
Vaccinated (at least one dose)	900	(67%)
Not vaccinated	393	(33%)

*Note*: Race/ethnicity question asked subjects to select all that apply.

### 2.2. Experimental design

All respondents were asked to evaluate a pair of hypothetical vaccines. As of the time our survey went into the field, the three COVID-19 vaccines authorized for emergency use in the United States were of two basic types: either a one-shot regimen based on viral vector technologies (i.e. the Johnson & Johnson/Janssen vaccine) or a two-shot regimen based on mRNA technology (i.e. the Pfizer/BioNTech and Moderna vaccines). Accordingly, our choice set asked subjects to choose between two hypothetical vaccines, Vaccine A and Vaccine B, with these fundamental characteristics.

Subjects were randomly assigned to one of five experimental groups via the Qualtrics survey platform randomization function. Respondents in the control group did not receive any information about efficacy. This allows us to estimate a baseline preference based solely on vaccine characteristics apart from efficacy. The four treatment groups provided subjects with information about each vaccine’s efficacy, but the treatments defined efficacy in different ways: efficacy in protecting against symptomatic infection; severe illness hospitalization and death; and all three combined (Table 2). The efficacy values on each dimension were drawn from real-world data about the Johnson & Johnson/Janssen vaccine (whose basic characteristics are the same as Vaccine A) and the Pfizer/BioNTech vaccine (whose basic characteristics are the same as Vaccine B). The first treatment defined efficacy as protection against symptomatic illness, with Vaccine A described as having 66% efficacy [30] and Vaccine B as having an efficacy rate of 91% [31]. The second treatment defined efficacy as protection against severe disease with Vaccine A reporting 85% efficacy [32] and Vaccine B 95% efficacy [31]. The third treatment reported that both Vaccines A and B were more than 95% effective against hospitalization and death. The final treatment reported all three efficacy measures for each vaccine.

**Table 2:** Experimental design.

	Control		Treatment 1		Treatment 2		Treatment 3		Treatment 4	
	*Vax A*	*Vax B*	*Vax A*	*Vax B*	*Vax A*	*Vax B*	*Vax A*	*Vax B*	*Vax A*	*Vax B*
Efficacy	--	--	66% against symptomatic illness	91% against symptomatic illness	85% against severe disease	95% against severe disease	> 95% against hospitalization and death	> 95% against hospitalization and death	66% against symptomatic illness	91% against symptomatic illness
									85% against severe disease	95% against severe disease
									> 95% against hospitalization and death	> 95% against hospitalization and death
# Doses	1 shot	2 shots	1 shot	2 shots	1 shot	2 shots	1 shot	2 shots	1 shot	2 shots
Technology	Viral vector	mRNA	Viral vector	mRNA	Viral vector	mRNA	Viral vector	mRNA	Viral vector	mRNA

*Note*: The attributes of Vaccine A mirrors those of the Janssen COVID-19 vaccine, while the attributes of Vaccine B mirrors those of the Pfizer COVID-19 vaccine.

All respondents where then asked, “if offered a choice between these two vaccines, would you choose Vaccine A, Vaccine B, or neither?” Reporting was guided by the CONSORT checklist (S2 Table).

### 2.3. Analytic strategy

The main quantities of interest in this study are the percentage of subjects choosing each vaccine, and the size of this relative preference gap across experimental treatments. T-tests are used to assess the statistical significance of the preference gap between the vaccines (i.e. the difference in means of those choosing Vaccine A vs. B) in each treatment. One-way ANOVA with a Bonferroni correction to account for the increased risk of Type I error when making multiple comparisons is used to assess whether the size of the preference gaps for Vaccine A vs. Vaccine B significantly varies across experimental treatment groups.

## 3. Results

**Fig 1:**
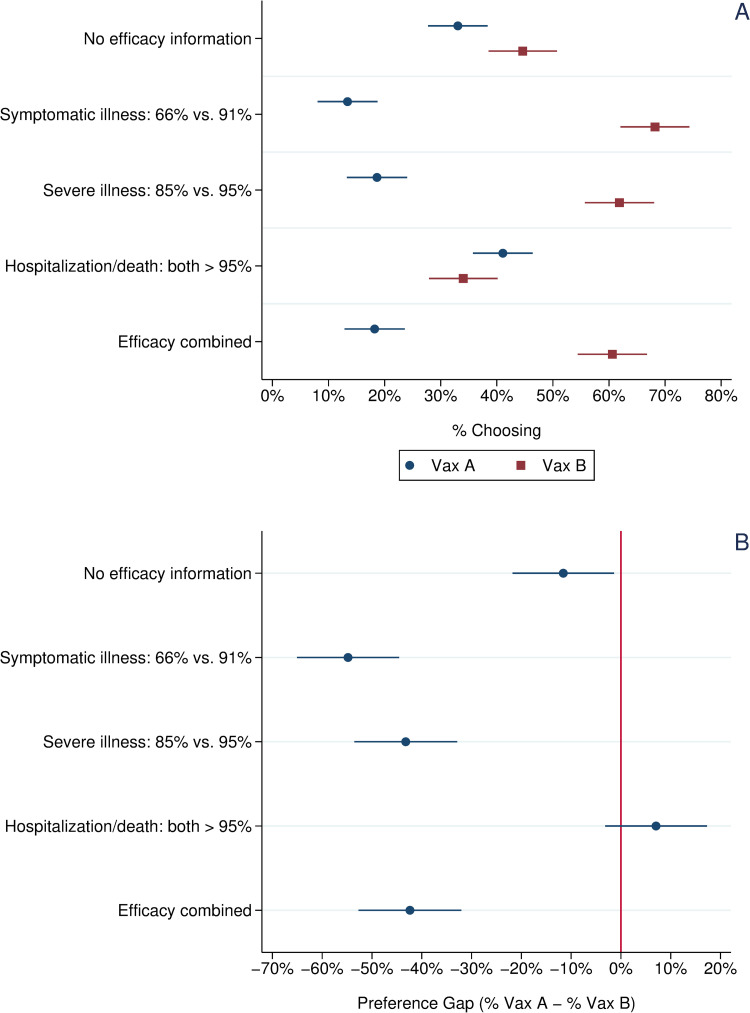
**Vaccine choice and preference gap across experimental treatments, all respondents.** *Note*: Panel A: Circles indicate the percentage choosing Vaccine A, a one-shot, viral vector vaccine with efficacy data mirroring that reported in the Janssen vaccine trials. Squares indicate the percentage choosing Vaccine B, a two-shot, mRNA vaccine with efficacy data mirroring that reported in the Pfizer vaccine trials. Panel B: Circles indicate the preference gap between Vaccine A and Vaccine B. Horizontal bars present 95% confidence intervals about each mean value.

Panel A of Fig 1 presents the percentage choosing Vaccine A (one-dose, viral vector) vs. Vaccine B (two-dose, mRNA) across the five experimental conditions among all respondents. Respondents in the control group who received no efficacy data had a slight preference for Vaccine B (45%) vs. Vaccine A (33%). This difference in means is statistically significant, p < .01, two-tailed test. As shown in panel B of Fig 1, this preference gap for Vaccine B widened dramatically in the first two treatment groups. The gap was widest (55%) in the treatment presenting efficacy data against symptomatic infection, but the preference gap remained large (43%) in the treatment group informed about efficacy against severe illness, even though the efficacy figures for the two vaccines, 85% vs. 95%, were much more similar than in the first treatment. An ANOVA with Bonferroni correction shows that this difference in the size of the preference gap across the two treatments is not statistically significant (p < .05, two-tailed test). In the third treatment group, where respondents were told that both vaccines were more than 95% effective in protecting against hospitalization and death, more subjects actually chose Vaccine B over Vaccine A (41% vs. 34%); however, the difference is not statistically significant (p = .11; two-tailed test). In the final treatment group, where respondents received all three efficacy figures for both vaccines, a sizeable preference gap again emerged with 61% choosing the Vaccine B versus only 18% choosing Vaccine A (difference in means is statistically significant, p < .01, two-tailed test). An ANOVA with Bonferroni correction shows that this preference gap, while significantly greater than that observed in the control and efficacy against hospitalization treatments, was not significantly different from the preference gap in either the efficacy against symptomatic infection or efficacy against severe illness treatments.

### 3.1 Unvaccinated respondents

**Fig 2:**
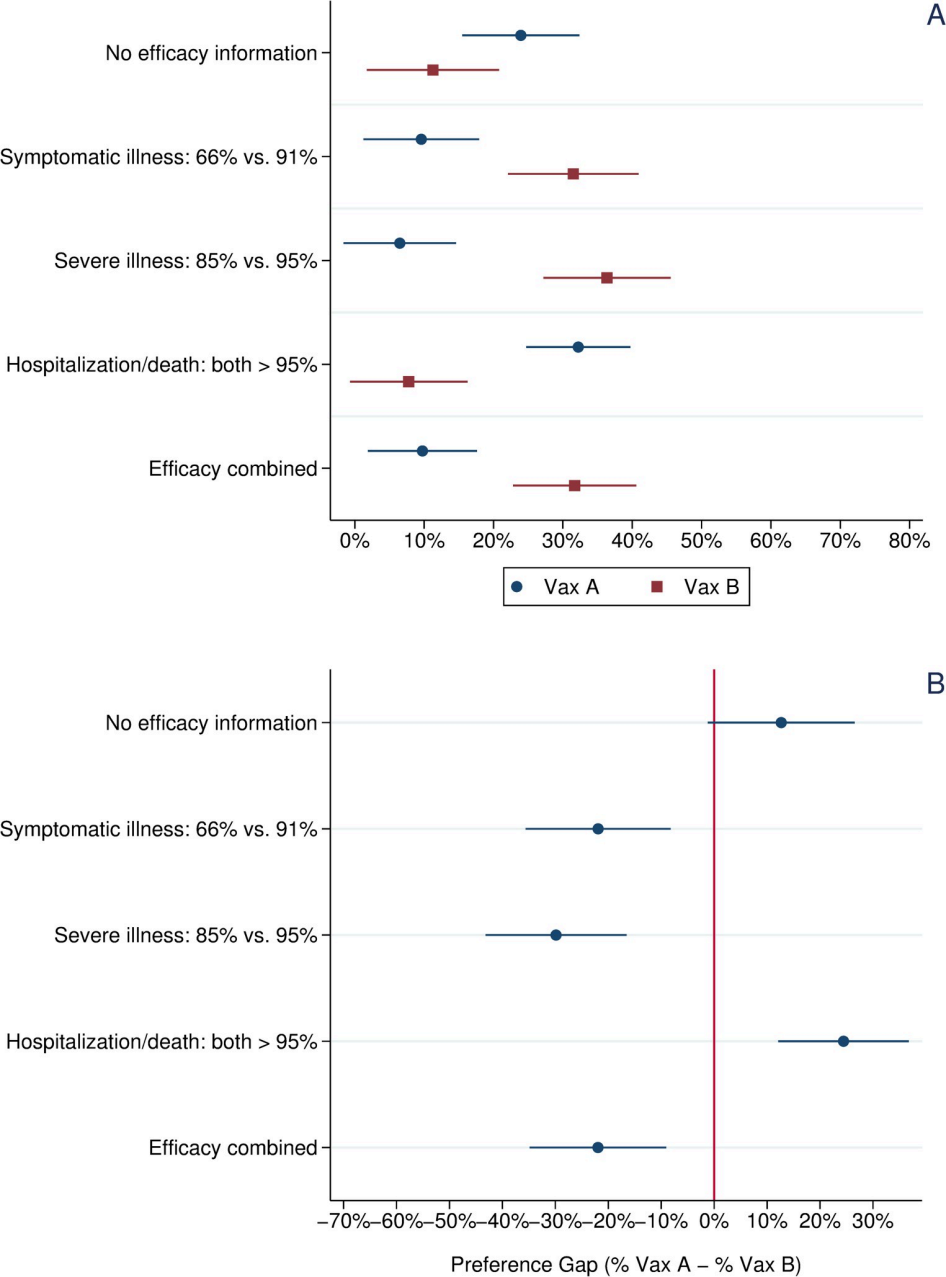
Vaccine choice and preference gap across experimental treatments, unvaccinated respondents. *Note*: Panel A: Circles indicate the percentage choosing Vaccine A, a one-shot, viral vector vaccine with efficacy data mirroring that reported in the Janssen vaccine trials. Squares indicate the percentage choosing Vaccine B, a two-shot, mRNA vaccine with efficacy data mirroring that reported in the Pfizer vaccine trials. Panel B: Circles indicate the preference gap between Vaccine A and Vaccine B. Horizontal bars present 95% confidence intervals about each mean value.

To examine more closely how efficacy framing may combat vaccine hesitancy, Fig 2 focuses specifically on the preferences of the 393 respondents who had not received a COVID-19 vaccine at the time of our survey. The sources of vaccine hesitancy are multiple, and our analysis does not seek to identify or disentangle those sources. However, understanding if and how those who were not vaccinated at the time of our survey responded differently to the efficacy frames is important for informing outreach efforts.

In the control group, unvaccinated respondents registered a slight preference for Vaccine A (24% vs. 11%); however, this difference in means narrowly misses conventional thresholds of statistical significance (p = .07, two-tailed test). In the treatments presenting Vaccine B’s higher reported efficacy figures against both symptomatic and severe infection, a significant preference gap emerged in the opposite direction with 22% more subjects choosing Vaccine B in the former condition and 30% more choosing Vaccine B in the latter condition.

In the treatment reporting only the two vaccines’ equivalent high efficacy marks in preventing hospitalization and death, the preferences of unvaccinated respondents reversed dramatically. In this treatment group, 32% chose the single shot Vaccine A versus just 8% who chose the two-dose Vaccine B regimen (this difference in means is statistically significant, p < .01, two-tailed test). However, in the final treatment presenting all three efficacy figures, a strong preference gap for Vaccine B re-emerged (32% choosing Vaccine B vs. just 10% choosing Vaccine A; this difference in means is statistically significant, p < .01, two-tailed test). Moreover, an ANOVA with Bonferroni correction shows that the size of this preference gap is statistically indistinguishable from that observed in the symptomatic illness and severe illness efficacy communication treatments.

### 3.2 Preferences of blacks and latinos

**Fig 3:**
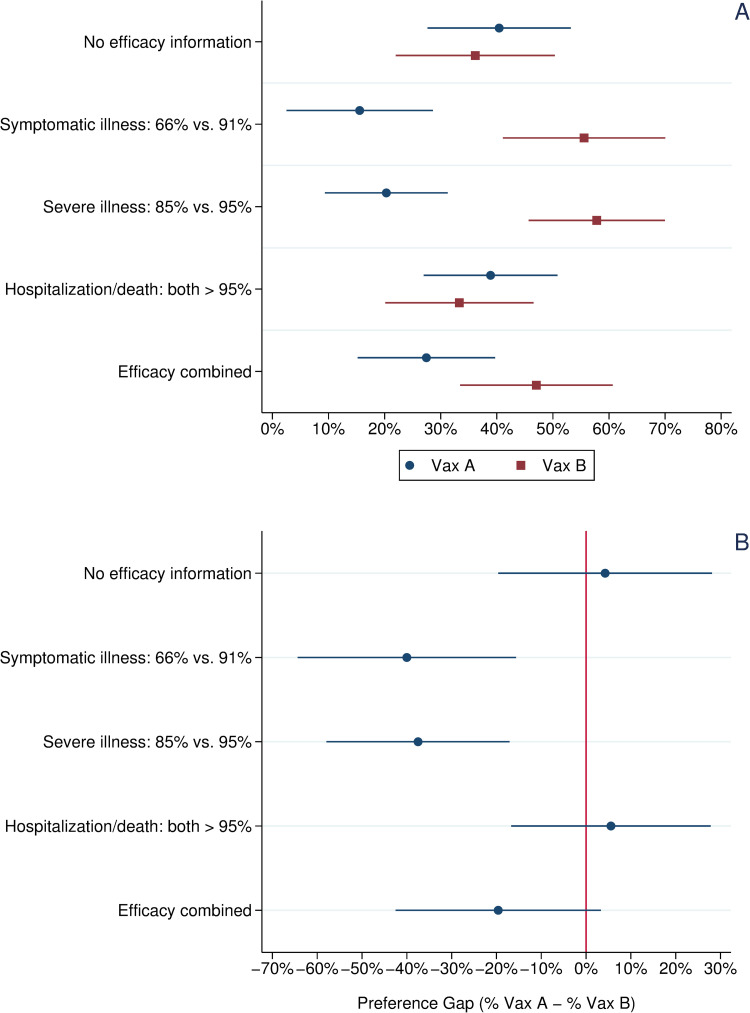
Vaccine choice and preference gap across experimental treatments, blacks and latinos. *Note*: Panel A: Circles indicate the percentage choosing Vaccine A, a one-shot, viral vector vaccine with efficacy data mirroring that reported in the Janssen vaccine trials. Squares indicate the percentage choosing Vaccine B, a two-shot, mRNA vaccine with efficacy data mirroring that reported in the Pfizer vaccine trials. Panel B: Circles indicate the preference gap between Vaccine A and Vaccine B. Horizontal bars present 95% confidence intervals about each mean value.

Our final analysis disaggregates the data by race/ethnicity to examine the preferences of whites vs. non-whites across treatments. Fig 3 focuses on the vaccine preferences of Blacks and Latinos, whom past research suggests have possess greater levels of vaccine hesitancy than whites, all else being equal [33–36]. S1 Fig presents a parallel figure for white respondents, which largely mirror the patterns for all respondents shown in Fig 1.

As shown in Panel A of Fig 3, in the control group Black and Latino respondents showed little preference between the two hypothetical vaccines. Just over 40% chose Vaccine A vs. 36% who chose Vaccine B; this difference in means is not statistically significant. By contrast, among non-Black or Latino respondents a significant preference gap for Vaccine B emerged in the control group (see S1 Fig). As observed in the aggregate (Fig 1), significant preference gaps emerged in the symptomatic and severe illness efficacy treatments with 40% more Black and Latino respondents preferring Vaccine B to Vaccine A in the former and 38% doing so in the latter. Again, following the pattern observed previously in the aggregate (Fig 1), this preference gap reversed in the treatment reporting that both vaccines were more than 95% effective in preventing hospitalization and death. In this treatment, more Black and Latino respondents preferred Vaccine A to Vaccine B (39% vs. 33%); however, the preference gap is not statistically significant. Finally, we observe an importance divergence in the combined efficacy treatment. When informed about both vaccines’ efficacy on all three metrics, more Black and Latino subjects preferred Vaccine B to Vaccine A (47% vs. 27%). This 20% preference gap is substantively smaller than that observed among Black and Latino respondents in the efficacy against symptomatic and severe illness treatments, though the differences in magnitude are not statistically significant. However, the 20% preference gap among Black and Latino respondents in this combined efficacy treatment is significantly smaller (p < .05, two-tailed test) than the 49% preference gap observed among non-Black or Latino respondents in the same treatment group (see S1 Fig)

## 4. Discussion

This study examines how communication about efficacy affects vaccine choice. Prior studies have found that public acceptance of COVID-19 vaccines increases [7,9,10] as reported efficacy rises and that efficacy is a major driver of choice between alternate vaccines [16,17,21]. However, all of the COVID-19 vaccines currently authorized for use have different estimated levels of efficacy from clinical trials or effectiveness from real world observational studies against different outcomes, for example against symptomatic infection, severe illness, hospitalization, or death. Moreover, emerging studies of efficacy against variants [37,38] has added even further nuance to public health communications about vaccine efficacy. As a result, it is critically important to explore how different ways of defining and communicating efficacy data to the public affect vaccine choice.

This study employed a survey experiment to examine how communications defining efficacy on different metrics affect vaccine choice. In the baseline control group that received no efficacy information, subjects expressed a slight preference for Vaccine B, a hypothetical two-dose, mRNA vaccine over Vaccine A, a one-dose, viral vector vaccine. However, unvaccinated subjects expressed a slight preference for the one-dose Vaccine A. The slight preference for Vaccine B in the aggregate may reflect the fact that most vaccinated Americans received either the Pfizer/BioNTech or Moderna vaccines. Similarly, the slightly greater preference for Vaccine A among the unvaccinated may reflect the lesser burden of a one-shot regimen. However, the differences are substantively small and largely mirror past research finding that the number of required shots and vaccine type have a relatively modest effect on vaccine choice [9,21,27,28].

The efficacy treatments significantly influenced choice between these two vaccines from the control group baseline. More respondents preferred the one-dose vaccine (Vaccine A) when the presented efficacy data emphasized both vaccines’ very strong protection against hospitalization and death. By contrast, in treatments defining efficacy as protection against symptomatic or severe illness, a significant preference gap emerged for Vaccine B, which boasted higher reported efficacy on these metrics. This suggests that even a relatively small difference in efficacy can have large effects on vaccine preferences. A similar pattern holds when looking at the preferences only of unvaccinated respondents (Panel B of Fig 2).

Among all respondents and unvaccinated respondents, a significant preference gap for Vaccine B also emerged in the combined efficacy treatment, which presented respondents with information about each vaccine’s efficacy on all three metrics. When subjects were informed only of both vaccines’ robust protection against hospitalization and death, this preference gap closed. However, adding this information to efficacy data showing Vaccine B’s stronger protection against symptomatic or severe illness did little to close the preference gap for Vaccine B except among Black and Latino respondents, suggesting that communicating efficacy just based on protection against symptomatic illness may undermine the appeal of a vaccine type (one-dose, viral vector) among a population that has been more vaccine hesitant than the white population.

As reports of breakthrough infections become more common [39], and the Omicron variant is proliferating [40], the way that medical and media professionals communicate information about the efficacy of existing vaccines [41] will take on even greater importance. In particular, invoking only one type of efficacy—for example against symptomatic illness versus severe outcomes such as hospitalization and death—may have the effect of discouraging vaccination among groups that might otherwise have a preference for the “less efficacious” vaccine. More broadly, our results indirectly suggest that public debates over efficacy against variants as well as changing vaccine efficacy level over time could have serious consequences for efforts to persuade the unvaccinated to be inoculated or reluctant individuals to get booster shots.

Our study has several limitations. First, while our quota-based sample roughly matches US Census demographics and compares favorably to other benchmark surveys (S1 Table), the percentage of our sample identifying as Latino is lower than US Census figures. Future research could replicate our analysis on a probability-based sample. An important limitation of our study design is that it was specifically constructed to examine how communications about efficacy affect choice between vaccines. Follow-on studies focused more specifically on the question of willingness to vaccinate can build on these findings. However, our findings suggest that the sometimes-conflicting information about vaccine efficacy against novel variants could undermine the campaign to increase vaccination rates. For example, much recent research [42,43] and corresponding media communications emphasizes that while currently available vaccines may be less effective in preventing symptomatic infection with the Delta variant, they continue to offer strong protection against hospitalization and death. While our study only examined the influence of similar messaging on vaccine choice, it is possible that communications pairing lower efficacy numbers with strong data on protection against hospitalization may be insufficient to boost confidence in vaccines among the hesitant. Future research should explore this question more directly.

The messenger may also matter, [20] and future research could explore how the cue-giver communicating efficacy information also shapes public vaccine attitudes and choice. Future research could also examine important sources of heterogeneity in response to communications about vaccine efficacy. For example, individual respondents’ varying levels of health literacy may significantly moderate the influence of efficacy communications on vaccine choice and willingness to vaccinate. Individuals with higher levels of health literacy may be better positioned to evaluate contrasting efficacy data against different health outcomes, while those with low health literacy may find it difficult to contextualize and interpret efficacy information. Better understanding such dynamics may play a key role in combatting vaccine hesitancy.

Finally, while this study focused exclusively on the effects of communications about vaccine efficacy, future research should also examine how such frames interact with other vaccine communications. For example, vaccine safety studies around the globe are tracking vaccine side effects [44–46], fears of which are a major source of hesitancy [7,9,10,15–17]. An emerging literature examines how the framing of side effect risks affects vaccine acceptance [47,48]. Future research could examine whether and how such frames interact to affect attitudes toward vaccination.

## Supporting information

S1 Table Comparative demographics. *Note*: Comparisons are made between our survey sample and the 2020 American National Election Study and 2018 General Social Survey. All Census figures taken from the 2018 American Community Survey.
(PDF)

S2 Table CONSORT checklist. (PDF)

S1 Fig Vaccine choice and preference gap across experimental treatments, not black or latino. *Note*: Panel A: Circles indicate the percentage choosing Vaccine A, a one-shot, viral vector vaccine with efficacy data mirroring that reported in the Janssen vaccine trials. Squares indicate the percentage choosing Vaccine B, a two-shot, mRNA vaccine with efficacy data mirroring that reported in the Pfizer vaccine trials. Panel B: Circles indicate the preference gap between Vaccine A and Vaccine B. Horizontal bars present 95% confidence intervals about each mean value.
(PDF)
