# Supplementary material for: Communication about vaccine efficacy and COVID-19 vaccine choice: Evidence from a survey experiment in the United States
Source: PLoS One. 2022 Mar 30;17(3):e0265011. doi: 10.1371/journal.pone.0265011 (PMC8967042; doi:10.1371/journal.pone.0265011)
Supplement: S1 Table — Note: Comparisons are made between our survey sample and the 2020 American National Election Study and 2018 General Social Survey. All Census figures taken from the 2018 American Community Survey. (PDF) [file pone.0265011.s001.pdf]

Supplementary Table S1: Comparative Demographics

	Lucid Sample	2020 ANES	2018 GSS	US Census
Demographics				
Black	13%	9%	16%	13%
Latino	9%	9%	6%	18%
Female	52%	54%	55%	51%
% College degree	38%	45%	33%	32%
Median age	43 years	52 years	48 years	38 years

Note: Comparisons are made between our survey sample and the 2020 American National Election Study and 2018 General Social Survey. All Census figures taken from the 2018 American Community Survey.
