# Supplementary figures and images for: Communication about vaccine efficacy and COVID-19 vaccine choice: Evidence from a survey experiment in the United States
Source: PLoS One. 2022 Mar 30;17(3):e0265011. doi: 10.1371/journal.pone.0265011 (PMC8967042; doi:10.1371/journal.pone.0265011)

A

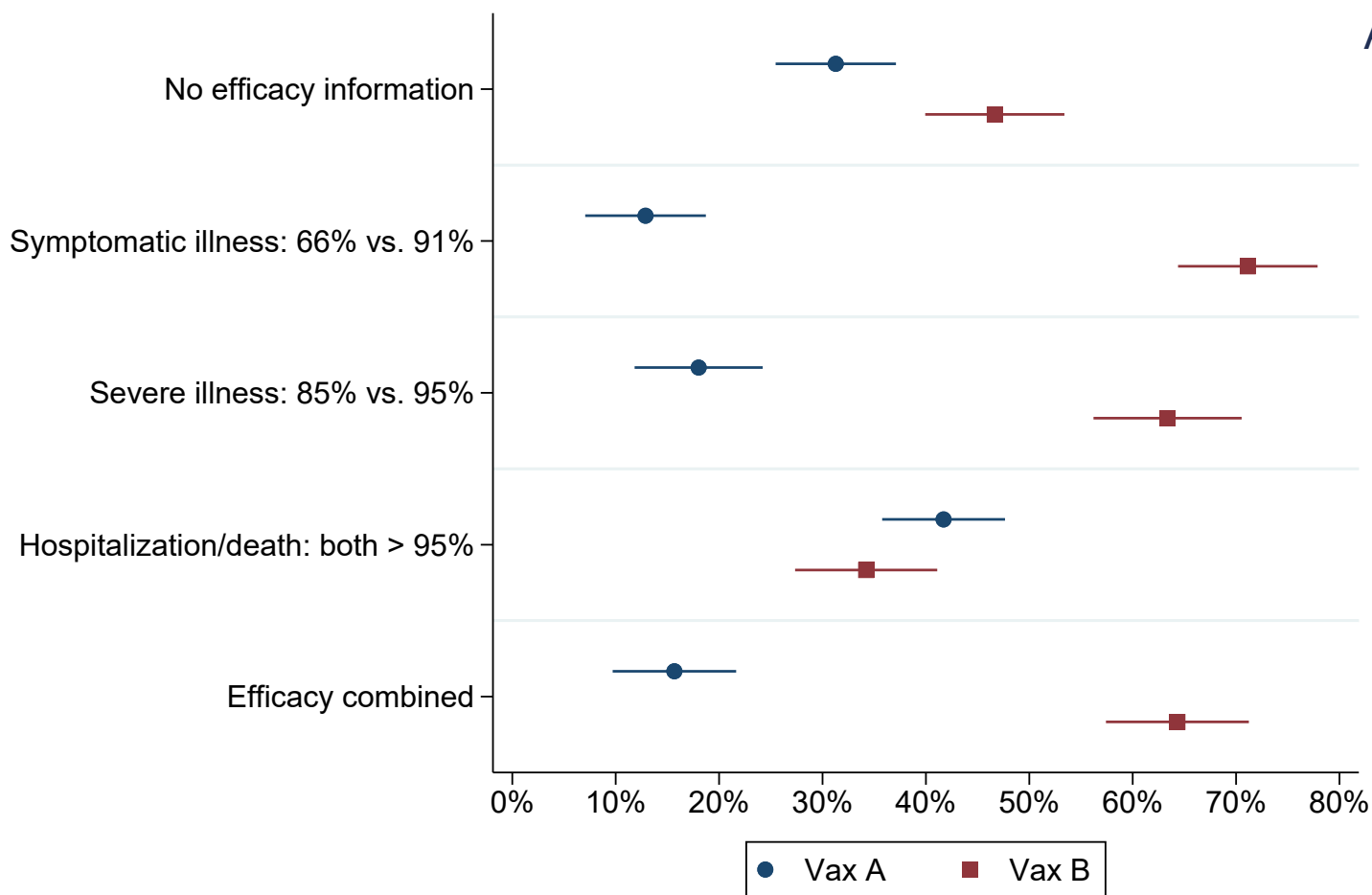

B

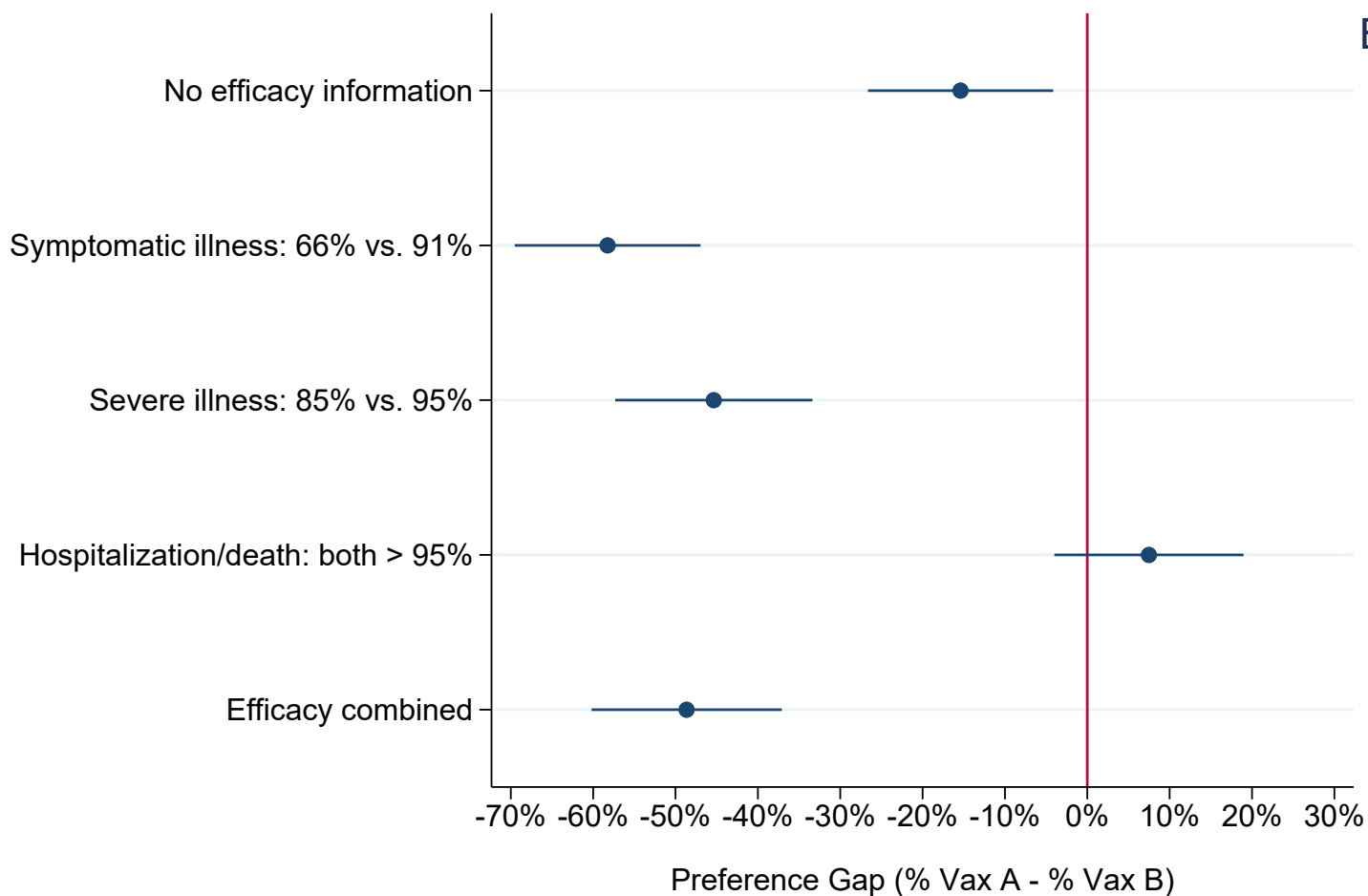

Supplement: S1 Fig — Note: Panel A: Circles indicate the percentage choosing Vaccine A, a one-shot, viral vector vaccine with efficacy data mirroring that reported in the Janssen vaccine trials. Squares indicate the percentage choosing Vaccine B, a two-shot, mRNA vaccine with efficacy data mirroring that reported in the Pfizer vaccine trials. Panel B: Circles indicate the preference gap between Vaccine A and Vaccine B. Horizontal bars present 95% confidence intervals about each mean value. (PDF) [file pone.0265011.s003.pdf]
